# Causes of suicidal behaviors in men who have sex with men in China: a national questionnaire survey

**DOI:** 10.1186/s12889-015-1436-8

**Published:** 2015-02-07

**Authors:** Hongquan Chen, Yang Li, Lixin Wang, Beichuan Zhang

**Affiliations:** Department of Dermatology, the Affiliated Hospital of Qingdao University, No.16 Jiangsu Road, Qingdao, Shandong 266003 China; Department of Dermatology, Qingdao Sanatorium of Shandong Province, No.16 Zhengyangguan Road, Qingdao, 266071 Shandong China; Sex Health Center, the Affiliated Hospital of Qingdao University, No.16 Jiangsu Road, Qingdao, Shandong 266003 China

**Keywords:** MSM, Suicide behaviors, Suicidal ideation, Risk factors, Questionnaire survey

## Abstract

**Background:**

Men who have sex with men (MSM) have become a high-risk group of HIV infection in China. To date, little is known regarding the psychological characteristics in Chinese MSM, especially the prevalence of suicidal ideation and attempts.

**Methods:**

A questionnaire survey was conducted on 2,250 MSM recruited from gay bars in 9 large cities in mainland China. Data on the prevalence of suicidal ideation and attempts as well as the underlying causes in the respondents were analyzed.

**Results:**

A total of 1530 MSM responded to the question regarding previous suicidal ideation and attempts. Of these respondents, 26.01% had ever considered suicide and 12.55% actually attempted suicide at least once. Romantic gay relationship breakup was the number one cause of suicide behaviors, followed by self-objection to homosexuality, difficulties in finding gay partners or in getting used to heterosexual marriage life, sudden emotional hurts from unexpected events, illegal status of gay marriage in China and disclosure of homosexuality.

**Conclusions:**

The survey study has led to a better understanding of the factors contributing to suicide behaviors of MSM in China, which may have significant implications in developing preventive strategies against suicide behaviors in this unique group of individuals.

## Background

The world has witnessed an economic miracle over the past three decades in mainland China. Parallel to the rapid economic growth, HIV/AIDS is increasingly become a public health problem in recent years in the country [1]. According to a recent report on the global AIDS epidemic from the Joint United Nations Programme on HIV/AIDS, currently there are 740,000 people living with HIV in China and approximately 26,000 HIV-infected Chinese residents died from AIDS in 2009 alone [2]. One of the most significant changes in the HIV epidemic in recent years in China is the emergence of men who have sex with men (MSM) as one of the subpopulation groups most at risk of HIV/AIDS [3,4]; HIV prevalence among MSM in large urban areas in China has reached a double-digit level [5].

MSM are more likely to develop various mental health problems [6,7], including suicidal behaviors than the heterosexual peers. Nevertheless, the severity of these problems varies considerably among geographic areas and racial and age groups [8]. In a telephone interview-based study conducted in the US, Paul and colleagues showed that 21% of 2881 urban MSM interviewed had planned to suicide, 12% attempted suicide at least once and 6% even made multiple attempts [9]. In another US study that aimed to compare the racial/ethical difference in homosexuality-associated mental disorders, black and Latino gay and bisexual men were found to have increased risk of suicide attempts as compared with White peers [10]. In a study on the mental disorders of Asian MSM using the Mini International Neuropsychiatric Interview criteria, 45% of 150 MSM in Mumbai, India had ever considered suicide while 29% experienced major depression and 24% experienced anxiety [11]. In China, our previous study showed that one third of MSM with high-risk behaviors in large cities had considered suicide [12].

The reasons for increased risk of psychological distress and suicidal behaviors in MSM are not fully understood. From the existing literature, it seems that the causes of suicidal behaviors in MSM may vary with race, age, education level, socioeconomic status, geographic area, etc., similar to the severity of mental disorders in MSM. To date, few studies have assessed the factors triggering suicide behaviors of MSM in China, although elevated suicidal intent in Chinese MSM has been consensually recognized. The present study was therefore undertaken to assess the risk factors for suicidal ideation and attempts in a purposive sample of MSM recruited from hidden gay bars in nine large cities in mainland China.

## Methods

### Questionnaire design

During the 10th Five-Year Plan (2001-2005) of the People’s Republic of China, the Chinese Ministry of Science and the Chinese Ministry of Health jointly launched and supported a National Key Research Project titled “Risk Analysis and Strategic Prevention of HIV Transmission from MSM to the General Population in China”. The core component of this project was a questionnaire survey conducted on MSM in hidden gay bars in nine large Chinese cities (i.e. Harbin, Shenyang, Xi'an, Zhengzhou, Shanghai, Nanjing, Wuhan, Chongqing, and Chengdu). The entire survey included a total of 214 questions generated in the Chinese language in a format of multiple choice or true/false option. Five of these questions were focusing on the suicidal aspect of the psychological and behavioral characteristics of MSM. These five questions are: 1) Have you ever considered suicide? 2) If yes, what are the reasons (a: self-objection to homosexuality; b: pressure from sexual orientation disclosure; c: pressure from forced marriage with a woman; d: inability to adapt to a marriage life; e: failure in attempts to find same sex partners; f: breakup of a romantic homosexual relationship; g: sudden emotional hurts due to cultural stigmas, prejudice and discrimination against homosexuality; h: illegality of same sex marriage; i: others)? 3) Have you ever attempted suicide? 4) If you have attempted suicide once, what was your age at that time and what was the most important underlying reason? 5) If you have attempted suicide more than once, can you specify how many attempts, ages at which these attempts were made and the most important reasons for these attempts? The respondents were allowed to make more than one choices when answering question number 2. The information on the HIV status of the survey participants was also collected.

### Survey execution

Given the sensitive nature of homosexuality, MSM in China are usually hard to be identified and probed. A snowball sampling approach was adopted to recruit subjects in this survey study. Through this approach, the subjects in the upper layer recruited potential subjects in the lower layer from their acquaintances.

Survey questions were printed in the Chinese language and distributed to the recruited subjects. Both partially and completely answered questionnaires were collected. The study protocol was critically reviewed and approved by the Institutional Ethics Committee of Qingdao University School of Medicine.

The execution of the survey strictly followed the ethical principles outlined in the Declaration of Helsinki regarding human clinical research. A written consent signed in person in the snowball sampling process was obtained from all subjects.

### Data categorization and analysis

A total of 2250 subjects returned their questionnaires. The subjects were categorized into two groups based on their answer to the first question in the survey: suicide intent and non-suicide intent (control). Those in the suicide intent group were stratified into a suicide attempt group and a non-suicide attempt group based on their answer to the third question. Accordingly, a mean and standard deviation (SD) of percentage were calculated for each of the categorical variables included in the study. Differences between groups were analyzed with chi-square test or Mann-Whitney Test. Correlations between the included variables and suicidal thoughts or suicide attempts were assessed by a multiple regression analysis. Differences were considered significant when P < 0.05.

## Results

Out of the 2250 respondents in total, 1530 responded to the first survey question (i.e., whether they had ever considered suicide) of the study. To this question, 398 (26.01%) respondents answered yes (i.e., they had ever thought about committing suicide) and the remaining 1132 (73.99%) respondents answered no (i.e., they did not consider suicide). The mean age of the 398 respondents who had suicide intent was 26 (23.0-33.5) years, not different from 26 (22-35) years in the 1132 respondents who did not have suicide intent (P > 0.05). The reasons for suicidal ideation in the 398 respondents who had considered are presented in Table 1. The most frequently answered reason was breakup of the romantic relationship with gay partners, followed by self-objection to homosexuality, failure to find appropriate gay partner(s), pressure from being forced to get married to a woman, difficulties in getting used to a marriage life, sudden emotional hurts from unexpected events, illegal status of gay marriage in China, and disclosure of sexual orientation. Please note: not all the 398 respondents who had suicide intent answered all the subquestions regarding the causes of suicide thoughts; this is why in the percentage calculation for some variables the denominator was 390 instead of 398.

**Table 1:** **Importance of major causes of suicide behaviors in MSM in China**

**Cause**	**N**	**n**	**Percentage**
Romantic gay relationship breakup	398	134	33.67%
Self-rejection of homosexuality	390	120	30.77%
Difficulty finding gay partner(s)	390	66	16.92%
Pressure from being pushed to get married	390	58	14.87%
Difficulty getting used to a marriage life	390	46	11.79%
Sudden emotional stress from unexpected events	390	45	11.54%
Lack of legislation of same sex marriage	398	44	11.31%
Disclosure of homosexuality	390	30	7.69%

Note: 398 out of the 1530 MSM who responded to the question regarding suicide behaviors had suicidal ideation, but not all of these 398 respondents equally answered each of the sub-questions regarding the individual causes of suicidal ideation. For this reason, the number of denominator in the percentage calculation was different for different causes. N: number of the MSM who responded to the specific cause in the first column; n: number of the respondents who had suicidal ideation due to the corresponding cause.

In the 1530 respondents who responded to the question regarding suicide behaviors, 192 (12.55%) actually attempted suicide. Attempt was made once, two times and more than two times in 142, 34 and 16 respondents respectively.

The HIV infection rate was 2.6% in the respondents who had suicide intent and 2.3% in those who had no suicide intent. The difference between these two groups of MSM was not significant (P > 0.05).

## Discussion

Suicide is the 13th leading cause of death worldwide [13] but is the 5th leading cause of death in China [14]; it is estimated that nearly half of the global suicide cases are from China, India and Japan. In the general population in China, the overall lifetime prevalence of suicidal ideation was 2.3% (2.8% in the rural sample versus 1.8% in the urban sample) and the prevalence of suicidal attempt was 1.0% (1.3% in the rural sample versus 0.9% in the urban sample) [15]. In the present survey, we found that 26.01% (398/1530) of MSM who responded to the question regarding suicide ideation reported suicide intent and 192 (12.55%) of these respondents had actually attempted parasuicide at least once. Apparently, the prevalence of either suicidal ideation or attempt in the unique population of MSM in China, is significantly higher than that in the general Chinese population; our finding is in agreement with the findings from studies in other regions of the world where homosexual and bisexual men are at a far greater risk of suicidal behaviors [6,9-11].

Suicide is a complex human behavior which is hardly predictable. Nevertheless, a variety of factors have been demonstrated to contribute to suicide. In this study, our survey analysis showed that romantic relation breakup with gay partner(s) was the single most important trigger of suicidal behaviors in MSM in China and 33.67% Chinese MSM involved in our survey had ever considered suicide when their romantic gay relationships were broken up (Table 1). This is in contrast to the results from a recent study on the causes of suicide behaviors in the general population in China where Marriage/Love (51.3%) after Family/Home (60.7%) and Health/Hospital (53.8%) was found to be the third most common categories of negative life events preceding suicide attempts in the rural Chinese youth [16]. Based on the traditional Chinese moral standard, gay, lesbian, bisexual, and transgender individuals as well as those who are sexually active are generally and collectively regarded as “dissolute” and “irresponsible” in China. Apparently, our finding suggests that MSM may be the same or even more serious about their romantic gay relationships as married heterosexual couples about their marriage instead of being “dissolute” and “irresponsible” as previously thought.

The second most important trigger of suicide behaviors in MSM in China as demonstrated in this study was the self-unacceptance of or self-objection to homosexuality. Culturally, strong stigma and discriminations exist against homosexual and bisexual behaviors in China, which is easy to understand. However, to our surprise, the survey data in this study showed that 120 (30.77%) out of the 390 MSM thought about suicide because they could not accept their homosexuality themselves. This finding implies that health professionals, social workers and all others including parents and relatives who do care about the well-being of MSM should pay more attention to the psychological health of this particular group of people to minimize their suicide attempts.

Other causes of suicide behaviors in the MSM surveyed in this study include difficulties in finding gay partners or in getting used to heterosexual marriage life, pressure from being forced to marry a woman, sudden emotional stress from unexpected events, illegal status of gay marriage in China and disclosure of homosexuality to others. All these causes have been also reported in various previous studies.

While elevated suicidal ideation in MSM has been reported to be associated with the positive HIV tests in some studies [17,18], Gibbie et al. has observed no positive correlation between psychological problems including suicide behaviors and HIV status in MSM [19]. Supporting the observation of Gibbie et al., we found no difference in the rate of HIV infection between MSM who had suicide behaviors and those who had no suicide behaviors (2.6% vs 2.3%, P > 0.05).

Sociodemographic data of the subjected analyzed in this study were presented in one of our previous papers [12]. In the present study, we did not include sociodemographic variables like income, education, profession, etc. in our analysis of reasons for suicide attempts in MSM in China. This is the major limitation of this study.

## Conclusions

Out of a total of 2250 MSM involved in this large-scale questionnaire survey conducted in 9 large Chinese cities, 1530 respondents answered the question regarding previous suicide behaviors. Of these respondents, 26.01% had ever considered suicide and 12.55% actually attempted suicide at least once. Romantic gay relationship breakup was the number one reason for the suicide behaviors, followed by self-objection to their homosexuality, difficulty in finding gay partners, difficulty in getting used to heterosexual marriage life, sudden emotional hurts from unexpected events, illegal status of gay marriage in China and disclosure of homosexuality. Identification of the factors contributable to suicide behaviors of MSM in China may have significant implications in developing preventive strategies against suicide behaviors in this unique group of individuals.
